# The association between cold extremes and neonatal mortality in Swedish Sápmi from 1800 to 1895

**DOI:** 10.1080/16549716.2019.1623609

**Published:** 2019-06-24

**Authors:** Lena Karlsson, Erling Lundevaller, Barbara Schumann

**Affiliations:** a Centre for Demographic and Ageing Research (CEDAR), Umeå University, Umeå, Sweden; b Department of Sociology, Umeå University, Umeå, Sweden; c Department of Epidemiology and Global Health, Umeå University, Umeå, Sweden

**Keywords:** Neonatal mortality, temperature, seasonality, indigenous population, preindustrial societies, Sweden

## Abstract

**Background**: Studies in which the association between temperature and neonatal mortality (deaths during the first 28 days of life) is tracked over extended periods that cover demographic, economic and epidemiological transitions are quite limited. From previous research about the demographic transition in Swedish Sápmi, we know that infant and child mortality was generally higher among the indigenous (Sami) population compared to non-indigenous populations.

**Objective**: The aim of this study was to analyse the association between extreme temperatures and neonatal mortality among the Sami and non-Sami population in Swedish Sápmi (Lapland) during the nineteenth century.

**Methods**: Data from the Demographic Data Base, Umeå University, were used to identify neonatal deaths. We used monthly mean temperature in Tornedalen and identified cold and warm month (5th and 95th) percentiles. Monthly death counts from extreme temperatures were modelled using negative binomial regression. We computed relative risks (RR) with 95% confidence intervals (CI), adjusting for time trends and seasonality.

**Results**: Overall, the neonatal mortality rate was higher among Sami compared to non-Sami infants (62/1,000 vs 35/1,000 live births), although the differences between the two populations decreased after 1860. For the Sami population prior 1860, the results revealed a higher neonatal incidence rate during cold winter months (<−15.4°C, RR = 1.60, CI 1.14–2.23) compared to infants born during months of medium temperature. No association was found between extreme cold months and neonatal mortality for non-Sami populations. Warm months (+15.1°C) had no impact on Sami or non-Sami populations.

**Conclusions**: This study revealed the role of environmental factors (temperature extremes) on infant health during the demographic transition where cold extremes mainly affected the Sami population. Ethnicity and living conditions contributed to differential weather vulnerability.

## Background

Studies in which the association between temperature and mortality is tracked over longer periods covering demographic, economic and epidemiological transitions are quite limited [1]. This is especially true when it comes to weather variations and neonatal mortality (usually defined as deaths during the first 28 days of life) [2–4] in pre-industrial sub-Arctic environments characterised by long and cold winters, and short and mild summers.

That the health of indigenous populations compares less favourably with their nonindigenous counterparts living in the same region area is known among both contemporary and historic populations. During the eighteenth century, the Sami in Swedish Sápmi (Lapland, the Sami’s traditional land) remained very isolated, partly based on the vast distances between households and the extreme cold of the area for much of the year [5]. Previous studies have found that Sami and non-Sami populations in Swedish Sápmi revealed different age-specific mortality patterns, life expectancies and infant mortality rates during the eighteenth and nineteenth centuries [5–7]. Generally, the infant mortality rate was higher among the Sami population compared to the non-Sami population until 1860, after which the differences decreased over time [5]. Comparing seasonal patterns of neonatal mortality during the nineteenth century, the mortality rate was higher during the winter for Sami and non-Sami populations [8]. How these seasonal patterns changed over time and in relation to temperature differences during the winter is still unknown.

Contemporary research into temperature extremes and infant mortality is generally concerned about hot extremes, whereas there is a lack of studies on the association between cold extremes and infant mortality. Studies conducted in contemporary societies have shown that extreme low temperatures are associated with higher infant mortality in Arctic populations [9] and with an increase in sudden infant deaths [10]. For historical populations, previous studies have identified socio-economic differences with regards to vulnerability to temperature extremes on infant and neonatal mortality, in which the effect of low temperatures was more pronounced among the lowest social groups [1,11]. However, differences in vulnerability to temperature extremes between ethnic groups living in the same area are still poorly understood.

### Research aim

The aim of this study was to analyse the association between extremely low temperatures and neonatal mortality among Sami and non-Sami populations in Swedish Sápmi during the nineteenth century (1800–1895).

## The impact of weather on neonatal mortality

From previous research on infant mortality in historical populations, we know that the season of birth affects the risk of infant mortality and season of birth is, therefore, a good proxy for living conditions *in utero* and during early infancy [8,12]. Neonatal mortality also appears to be associated with the seasonality of infectious diseases and extreme temperatures, e.g. low winter temperatures [13] or high summer temperatures [14].

Factors linking weather conditions to seasonal variations in birth outcomes have been observed, such as an increased risk of preterm birth, low birth weight and stillbirths following temperature extremes [15,16]. Weather extremes also influence maternal health, nutritional status and workload during pregnancy [15,16]. Regarding season and extreme temperatures, different patterns have been revealed across locations. In nineteenth-century Italy and Spain, neonatal mortality was highest among winter-born infants. This was explained by the cold winter climate and high risk of respiratory infections during the first sensitive weeks of life [17,18]. The high level of winter neonatal mortality in Veneto, Italy, from 1750 to 1850 was mainly found to be the consequence of maternal malnutrition and low birth weight which, in turn, amplified the effects of cold temperatures, either as the vulnerability of newborns to survive low temperatures and/or the parent’s inability to protect their infants from the cold [17]. In contrast, in nineteenth-century Russia and Poland, neonatal mortality was higher among infants born in the summer, explained as a combination of exposure to high temperatures and a higher risk of contracting infections of the digestive tract [19,20]. The higher risk among summer-born infants in Russia and Poland also related to socio-economic factors, in which mothers were expected to participate in farm work, thus leaving home and taking less care of their infant [19,20]. In Switzerland and the Netherlands, an almost non-existent difference was found between winter born and summer born, implying that infants were well cared for despite season-related difficulties [19].

Although previous studies have revealed the influence of mainly cold weather and neonatal mortality, studies that include temperature data are quite limited. In a previous study of age-specific mortality between 1749 and 1859 in the parish of Skelleftea (part of northern Sweden), we found that warmer winters were associated with lower mortality among adults and older children, but not among infants <1 year [21]. Following previous research on the association between climate factors (temperature) and infant mortality, the association is perceived as being different during early infancy (neonates) compared to later stages of infancy [16]. For example, winter constitutes a dangerous season for neonates but also for summer-born infants when entering their first winter [8,18].

One factor contributing to environmental vulnerability is artificial feeding as it influences both nutritional intake and exposure to diseases, especially during the summer with higher temperatures [16]. In nineteenth-century Sweden, mixed feeding or artificial feeding (cow’s milk) were common, especially in towns and on the northern coast [22,23]. However, in the northern hinterland, breastfeeding was widely practised and, for the Sami, breastfeeding was the only option. According to the clergy and physicians, Sami mothers breastfed their children for 2–4 years [24]. We believe that the risk of contamination and infectious diseases following artificial feeding was lower in Sápmi compared to the rest of Sweden, especially for the Sami population. It was only during the first days after birth, before the mother was able to produce milk, that Sami infants were given reindeer fat, which may have increased the risk of infection.

Studies on the effect of temperature extremes on infant mortality have revealed that populations adjust to the local climate and that mortality peaks in winter were more common in areas with milder winter temperatures compared to areas with harsher winter temperatures [16]. Many Sami became reindeer herders in the early seventeenth century, and the reindeer-herding year was traditionally divided into eight different seasons that corresponded to the shifting reindeer-herding conditions and work intensity [7]. We can assume that the Sami adjusted to the harsh climate to a higher extent compared to the settlers. However, the traditional nomadic lifestyle of the Sami contributed to the high neonatal mortality rate because after giving birth during migration, mothers only had a couple of days before moving on to the next settlement [25,26].

Another mechanism that could link cold weather conditions with neonatal mortality is the disease panorama in northern Sweden. Previous research on the association between season, temperature extremes and neonatal mortality among historical populations has mainly been conducted in Italy and Spain [11,17–19,27–29]. These countries have longer hotter summers and shorter milder winters, whereas northern Sweden has longer colder winters and shorter cooler summers. Compared to other studies into weather extremes and neonatal mortality, extreme heat is uncommon in northern Sweden and daily maximum temperatures rarely exceed 25°C, whereas daily minimum temperatures below −15°C are very common in winter. Generally, the meteorological winter (daily average temperature below 0°C) in Sápmi is long and extends from November to April in Southern Sápmi and October to April in northern Sápmi [30]. We can also assume that respiratory infections that are more common during the winter months will increase the mortality risk of infants being born during cold periods more pronouncedly in Sápmi compared to the Mediterranean countries.

## Methods

### Population data

**Figure 1.**
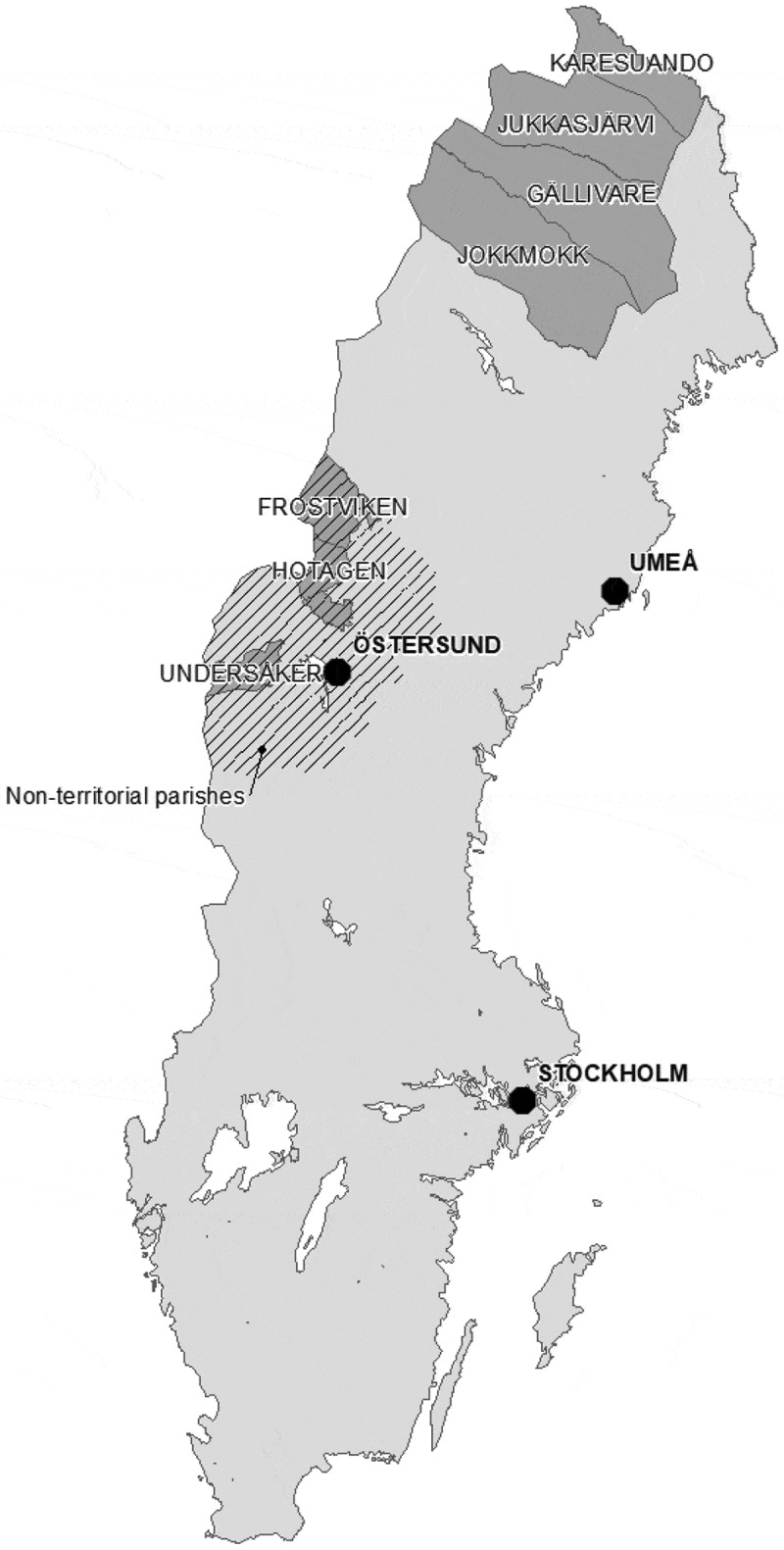
Map of Sweden, including parishes in the study area of Swedish Sápmi 1800–1895. Reproduced with permission from the Swedish National Archives.

The numbers of neonatal deaths based on month of birth and ethnicity were collected from 12 parishes (see Figure 1) in the Sápmi region during the nineteenth century (1800–1895). The data are from the POPUM database and comprise parish records (records of births, deaths, marriages and migration, as well as catechetical examination registers) that have been digitalized and linked by the Demographic Data Base, Umeå University [31].

The main variable, neonatal mortality, was defined as live born deaths that occurred during the first 28 days of life. Historical data such as this generally lack registered information of the date of birth and date of death of every single individual. Also, the cause of exit from the registries was sometimes unknown (unreported out-migration or unreported death). In a previous study, we found that a higher proportion of Sami ended their registration without a stated reason, mainly due to a higher out-migration resulting from difficulties related to reindeer herding [32]. It is possible that neonatal mortality is under-reported to a higher extent than mortality for other age groups because of the short duration (weeks) after birth [33]. Since the number of days of life is crucial to these analyses, only infants with a known birth and death date were included in the analyses. Around 92% of the population have both a known birth and death date in the data. Further, we have restricted our analyses to those infants born in the study area (Sápmi).

During the study period from 1800 to 1895, delivery of infants was a private matter (in one’s home) and birth practices differed between regions and populations. The Sami rarely had any contact with district physicians and midwives did not become established in this northern region until the late nineteenth century [5]. Because of their experience of reindeer herding, Sami men often assisted as midwives during delivery. Since the delivery of infants was not yet located in health centres, the data lack information of factors important to neonatal health and mortality, such as premature birth, birth weight and congenital defects [16,34]. During this period (1800–1895), information about the cause of death was poorly reported, especially for infants. From 1831, the clergy were no longer required to enter the cause of death in their records, with the exception of deaths related to smallpox, suicide, accidents, childbirth and major epidemics (SCB 1999).

**Figure 2.**
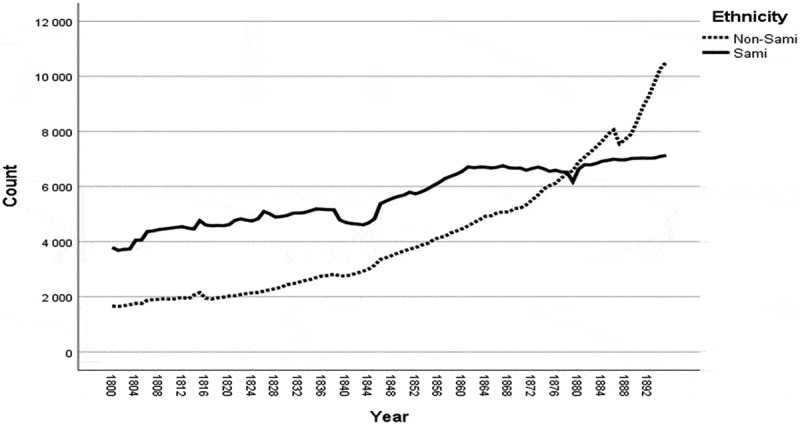
Sami and non-Sami populations in Sápmi from 1800 to 1895. Data: Demographic Data Base, Umeå University.

Ethnicity was not registered in church records, but the records provide opportunities to find information about a person’s ethnicity. A system of ethnic indicators that distinguish between the indigenous Sami population and the non-Sami population has been developed and added to the database by historian Gabriella Nordin [35]. The indicators of ethnicity are occupation, mortality, geographical information, name and family relations (if the individual had Sami parents, grandparents or siblings). Inclusion of the word ‘Lapp’, ‘Lappish’ or ‘Nomad’ is the most prominent indicator of Sami ethnicity to be found in the different sources (parish records) [5,35]. The non-Sami group includes the settlers who began moving to the Sami region from the mid-eighteenth century, mostly from the north coast of Sweden, Norway and Finland [5]. It was not until the mid-nineteenth century that a major increase in the non-Sami population occurred. From around 1880 onwards, the ethnic balance in Sápmi changed. This turned the Sami from a majority into a minority population in their own lands (Figure 2).

### Temperature data

Regular recordings of daily temperature and precipitation started in Sweden around 1860, although single weather stations had already been installed in the 1700s. We have records of temperature either as validated data from weather stations in northern Sweden or as reconstructed data based on measurements further south. Here, we used the monthly mean temperature in Tornedalen (northern Lapland) from January 1802 to December 1895, validated and homogenized by Klingbjer and Moberg [36]. From January 1800 to December 1801, we used the monthly average temperature in Umeå estimated from measurements in Uppsala and Tornedalen. We have used these temperature data in previous studies of mortality in northern Sweden during the nineteenth century [21,37]. All temperature values are given in degrees Celsius (°C). As a cutoff for low and high temperatures, we used the 5^th^and 95^th^ percentile of all monthly temperatures, respectively. For the present analyses, we defined temperature extremes based on the mean monthly temperature as cold (temperature at month of birth lower than −15.4°C), lagged cold (if the temperature in one of the three months prior to birth was lower than −15.4°C), warm (temperature in month of birth higher than 15.1°C) and lagged warm (temperature in one of the three months prior to birth higher than 15.1°C).

### Data analysis

The association between monthly death counts and temperature extremes was modelled using negative binomial regression accounting for over-dispersion. In order to check the model assumption, we used a likelihood ratio test between a negative binomial regression model and a Poisson regression model that revealed that the negative binomial model was more appropriate compared to the Poisson model. Negative binomial regression is a generalization of Poisson regression that allows over-dispersion [38,39]. To control for long-term trends in neonatal mortality and seasonal patterns, each model included a seasonal time trend variable. This variable is used to adjust the offset variable in the regressions to reflect the time-varying intensity of mortality. As a first step, the seasonal time trend variables were created by the Seasonal-Trend Decomposition Process Based on Loess (stl-function in R from package stlplus with parameters s.window = 24, t.window = 120, robust = TRUE), composing the time monthly mortality rate into a trend and season [40,41]. As a second step, fitted values (season + trend) and rates were created (fitted values/mean (fitted values)) for the Sami and non-Sami population, respectively. In the negative binomial regression models, the number of live births was added as an offset adjusted by the rates created in the second step (number of live births*rate).

We computed relative risks (RR) with 95% confidence intervals (CI), adjusting for time trends and seasonality. First, we conducted analyses of the association between temperature extremes and neonatal mortality for the Sami and non-Sami population separately. Second, we concentrated our analysis on births in the coldest months, December–February stratified by period. The 1860s has been revealed as a turning point where the overall mortality and life expectancy among the Sami improved and the difference in infant mortality rate between the Sami and the non-Sami decreased [42]. In this second step, we analysed winter neonatal mortality (December–February) over the two sub-periods: 1800–1859 and 1860–1895. In order to determine if temperature itself, overall, is statistically significant, we compared models with and without temperature extremes (warm/not warm, and cold/not cold) using ANOVA.

All analyses were conducted using R statistical software (version 3.4.3) with the MASS, season, stats, ggplot2 and stlplus packages.

### Ethical considerations

Ethics in research related to indigenous populations have been widely discussed [43]. Sweden still lacks ethical guidelines that are specifically aimed at Sami research. In this project, with its historical and long-term perspective of temperature and neonatal mortality, all individual information was anonymized.

## Results

### Descriptives of neonatal mortality and temperature in Swedish Sápmi from 1800 to 1895

**Figure A1.**
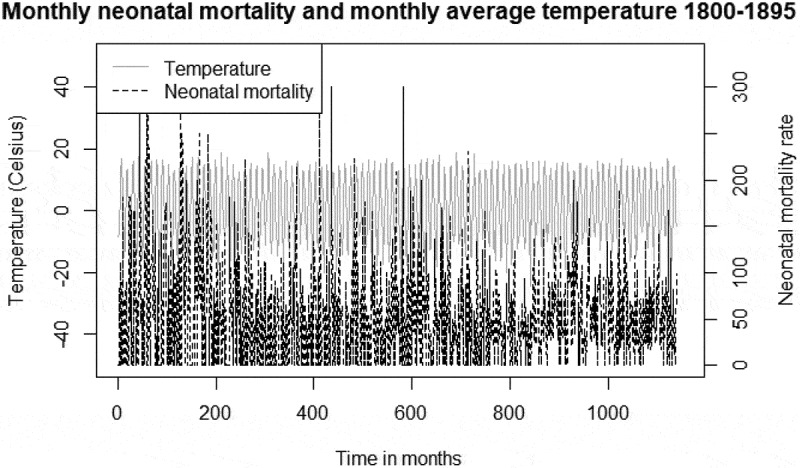
Monthly mortality and monthly mean temperature, 1800–1895. Data: Demographic Data Base, Umeå University and Klingbjer and Moberg (32).

Table 1 shows the descriptive statistics of mortality and temperature data. The total number of neonatal deaths during the entire period, 1800–1895, was 1,514. With regards to temperature, Table 1 reveals that the coldest month observed was found during the period 1860–1895, whereas there was a higher number of cold extreme months during 1800–1859. During the entire study period, 66 months were associated with cold extremes (≤-15.4°C), and these cold extremes were found in the months of January and February. With regards to maximum monthly mean temperatures, there was less variation between the sub-periods. In the Appendix, Figure A1 shows the monthly neonatal mortality rates and monthly average temperatures for the period 1800–1895.

**Table 1.** Descriptive statistics of mortality and temperature data, 1800–1895.

	1800–1859	1860–1895	Total
Number of observations (months)	720	432	1152
Number of live births	12,487	16,457	28,944
Number of neonatal deaths	654	860	1,514
Mean number of neonatal deaths per month	0.9	2.0	1.3
Percentage of neonatal deaths by ethnicity			
Sami	79.0	46.5	60.7
Non-Sami	21.0	54.5	39.3
Mean temperatures (°C)			
Monthly mean	0.3	0.3	0.3
Monthly minimum	−22.7	−24.3	−24.3
Monthly maximum	18.8	18.5	18.8
Number of warm/cold months			
Warm months (≥15.1°C)	34 (4.7%)	21 (4.9%)	55
Cold months (≤-15.4°C)	40 (5.6%)	16 (3.7%)	56

### Neonatal mortality from 1800 to 1895

**Figure 3.**
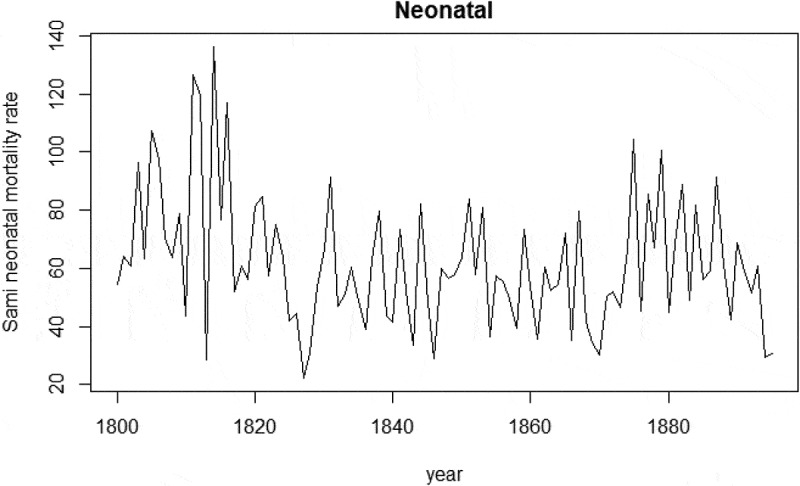
Sami neonatal mortality rate (deaths/1,000 live births), 1800–1895. Data: Demographic Data Base, Umeå University.

**Figure 4.**
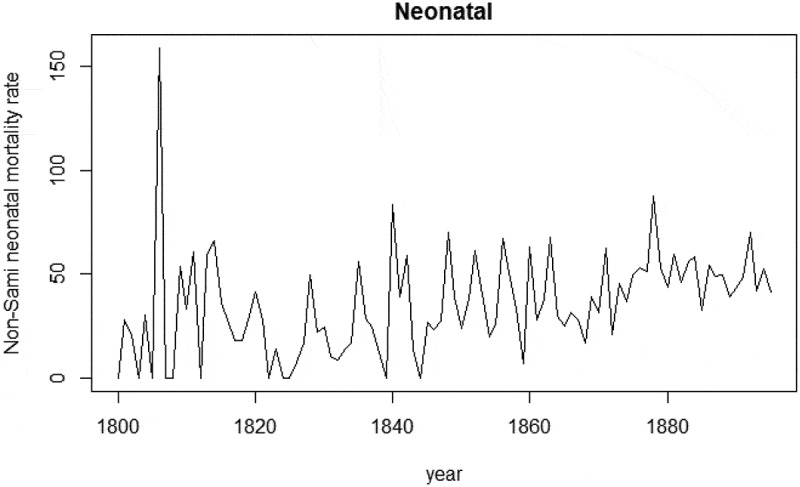
Non-Sami neonatal mortality rate (deaths/1,000 live births), 1800–1895. Data: Demographic Data Base, Umeå University.

From 1800 to 1895 the average neonatal mortality rate in Sápmi was 52/1,000 live births. Generally, the neonatal mortality rate was higher among the Sami compared to the non-Sami population, 62/1,000 and 35/1,000, respectively. For both populations, there were more fluctuations in neonatal rates during the first half of the nineteenth century compared to the second half of the century (Figures 3 and 4). This is particularly evident among the non-Sami population and corresponds to the population increase from the mid-nineteenth century onwards. From the 1870 onwards, the neonatal mortality rate among the non-Sami population was about 50/1,000, whereas the neonatal mortality rate among the Sami fluctuated between 50/1,000 to above 100/1,000.

**Figure A2.**
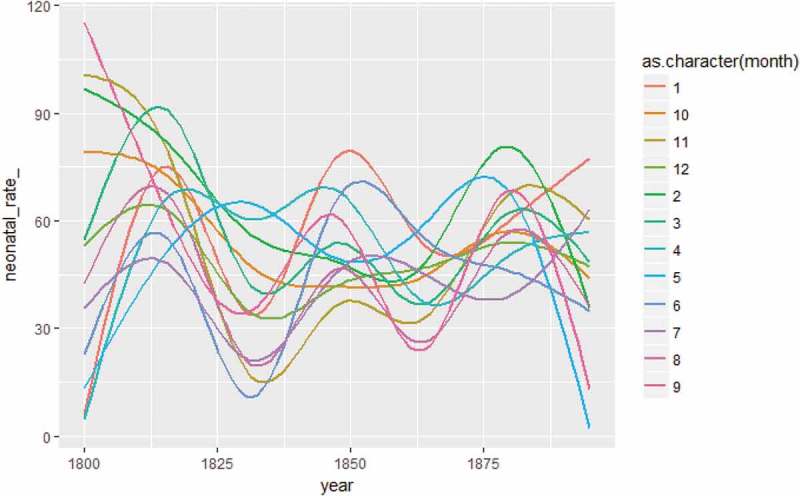
Neonatal mortality rate (smoothed) by month of birth, 1800–1895. Data: Demographic Data Base, Umeå University.

**Figure A3.**
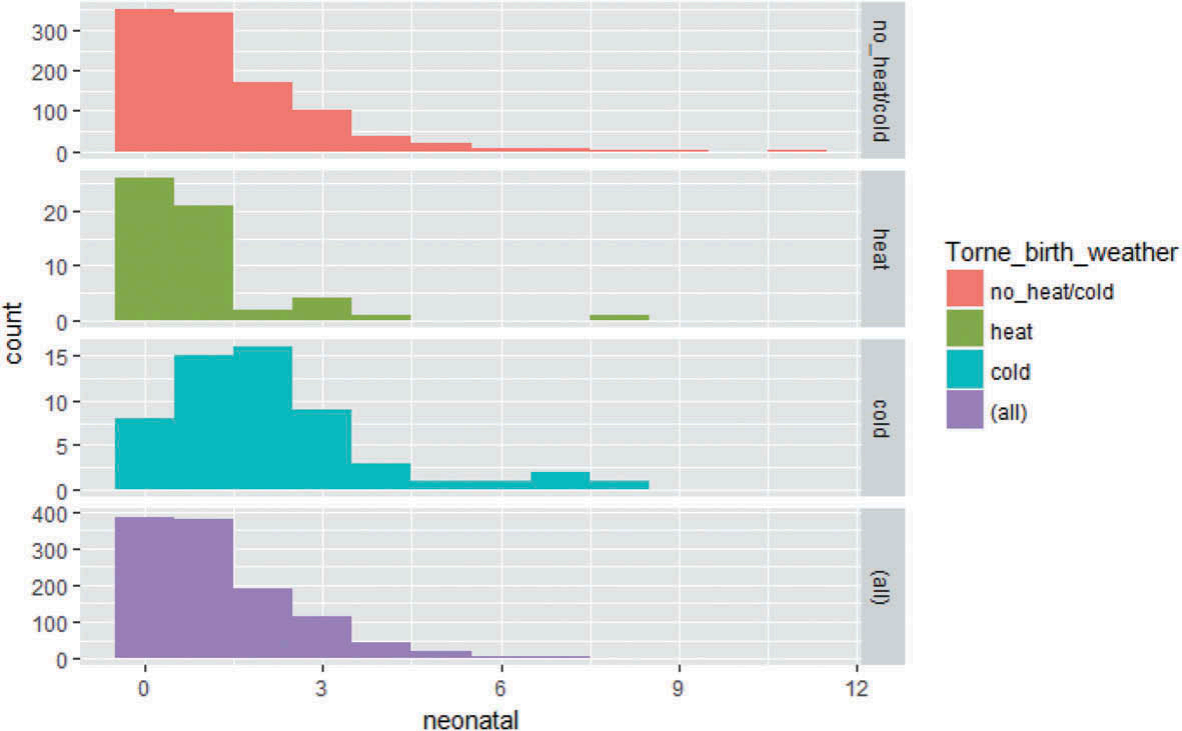
Distribution of neonatal deaths counts by warm months, cold months, average months and all months. Data: Demographic Data Base, Umeå University.

Figure A2 (Appendix) shows the seasonality of neonatal mortality decreased during the mid-nineteenth century (when neonatal mortality rates were generally low) and increased again at the end of the century. Figure A3 (Appendix) shows the distribution of neonatal death counts during warm and cold extremes for the entire period from 1800 to 1895, revealing that more deaths occurred during cold months and that the dispersion of deaths was greater during cold extremes compared to their warmer counterparts.

**Figure 5.**
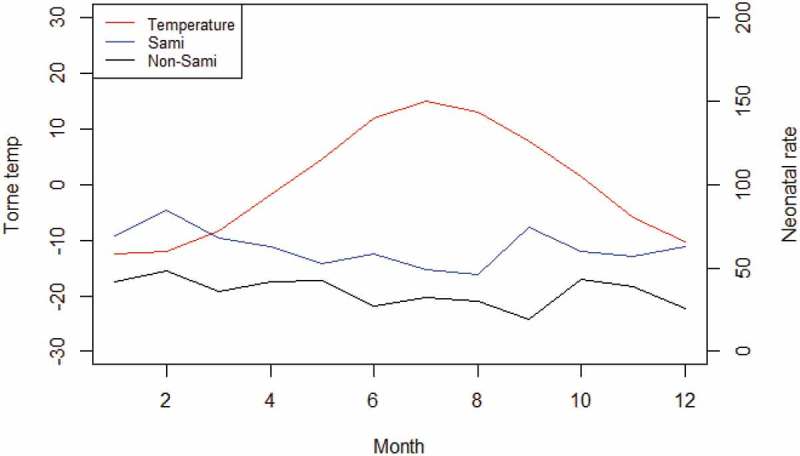
Mean Sami and non-Sami neonatal mortality rate and mean temperature by month from 1800 to 1895. Data: Demographic Data Base, Umeå University and Klingbjer and Moberg [36].

Continuing with the association between neonatal mortality and season of birth, Figure 5 reveals the mean neonatal mortality rate and mean temperature by month of birth from 1800 to 1895. Neonatal mortality rates were generally higher during the two coldest winter months of January and February, 61/1,000, and lowest during the summer. For Sami infants born from January–February, the neonatal rate was 76/1,000, compared to 45/1,000 among non-Sami infants. With the exception of the winter months, the neonatal mortality rate peaked in September for the Sami population, whereas September had the lowest neonatal mortality rate for the non-Sami population. For the non-Sami, this low neonatal mortality in September was followed by a peak in October, almost in parity with February.

### Neonatal mortality and temperature extremes

Table 2 shows that the relative risk of neonatal death among Sami infants born in cold months was 1.26 (95% CI 1.00–1.58), compared to infants born during months of no extreme cold. The relative risk can here be interpreted as the incident rate, 1.26 times the incident rate of infants born during months of no extreme cold. Even being born in warm months indicated a higher risk of Sami neonatal death (RR 1.21) although the confidence interval was large (0.70–1.78). For non-Sami neonatal mortality, we found no effect either of cold months or warm months. The chi-square ANOVA-test comparing the model with and without temperature at birth/prior birth for Sami revealed that temperature overall was not statistically significant, implying the need to carefully interpret the association between weather extremes and neonatal mortality from this model. In order to further investigate the association between cold extremes and neonatal deaths, the next model only included infants born during the cold winter months of December–February in the sub-periods 1800–1859 and 1860–1895. This analysis included 3,542 winter-born infants and 199 deaths from 1800 to 1859, as well as 4,482 winter-born infants and 265 deaths from 1860 to 1895.

**Table 2.** Relative risk of neonatal mortality by temperature extremes at lag 0 and lag 1–3 months with different time lags controlled for seasonal time trend, per ethnicity from 1800 to 1895.

	Sami RR (95% CI)	Non-Sami RR (95% CI)
Temperature at birth		
Warm	1.21 (0.78–1.78)	1.03 (0.67–1.50)
Cold	**1.26 (1.00–1.58)**	1.09 (0.77–1.51)
Temperature lag 1–3 months		
Warm	1.14 (0.92–1.39)	1.07 (0.82–1.37)
Cold	1.03 (0.86–1.23)	0.91 (0.69–1.17)

Models include controls of long-term trend and season. Cold and warm temperatures were not adjusted for each other. Bold numbers indicate statistical significance at the 5% level.

**Figure 6.**
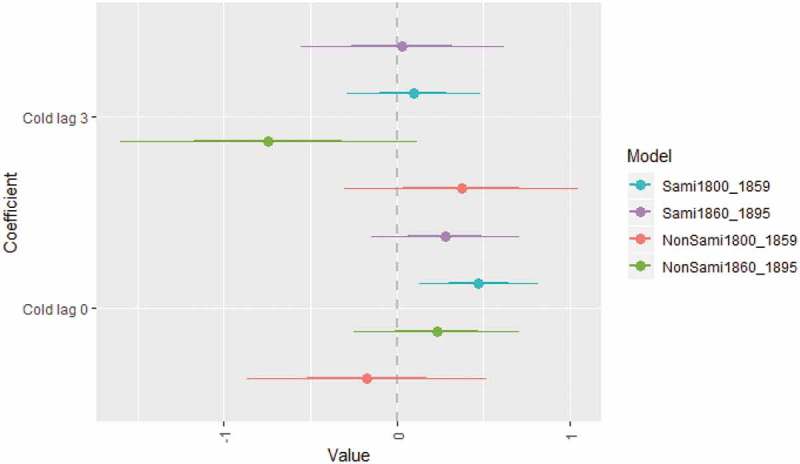
Regression coefficients of negative binomial regression between neonatal mortality and extreme low temperature from December to February, with time lags, controlled for seasonal time trend. Data: Demographic Data Base, Umeå University.

With the exception of cold extremes for the non-Sami population from 1800 to 1859, the estimates in Figure 6 point in the same direction, indicating a higher risk of neonatal mortality during extreme cold winters (December–February), although some confidence intervals were large. Exposure to extreme cold in winter was associated with increased neonatal mortality among the Sami during the period 1800–1859 (RR 1.60, CI 1.14–2.23). The chi-square ANOVA-test comparing the model with and without temperature at birth/prior birth revealed that temperature overall was statistically significant for Sami neonatal mortality during the period 1800–1859. No association between cold extremes and neonatal mortality was found for Sami 1860–1895 or for non-Sami in neither period.

## Discussion

During the nineteenth century, the neonatal mortality rate in Sápmi was 52/1,000 live births. The neonatal rates found in this rural area correspond quite well with the rates revealed by previous research on rural Sweden during the nineteenth century [7,33]. Although there are data for infant mortality (<1 year of age), there are no data specifically on the neonatal mortality rate in Sweden as a whole for the entire period of 1800–1895. Official statistics from 1860/1866 and 1891/1900 reveal that the neonatal mortality rates (in these historical statistics, defined as death within the first 30 days) were 47 and 34 out of 1,000 live births, respectively [44]. The neonatal mortality rates presented in this article are lower, or much lower, compared to other historical populations. For example, in Italy, the neonatal mortality rate during the nineteenth century was almost twice as high [11] as the neonatal mortality rate in Sápmi.

In our study population, neonatal mortality was almost twice as high among Sami compared to non-Sami infants from 1800 to 1895 (62/1,000 vs. 35/1,000 live births). Similar to infant mortality rates [5], the differences in neonatal mortality rates between the Sami and the non-Sami population decreased after 1860. During the nineteenth century, the population increased in the Sápmi region, with a major increase in the number of settlers from the mid-nineteenth century, turning the Sami from a majority into a minority population. The first settlers who moved into the Sápmi region were mainly adult men and older individuals, and family formation was delayed. The rapid increase in the non-Sami population, family formation and fertility from the mid-nineteenth century onwards is reflected in the high percentage of all neonatal deaths from 1860 to 1895.

We found a moderate U-shaped pattern in seasonality, with higher neonatal mortality rates for both populations during the winter. For the Sami population, the results revealed a higher neonatal incidence rate during cold months below −15.4°C. The extreme cold winter had a greater impact on Sami infants until 1860, seen as higher risk of neonatal deaths during colder months (RR = 1.60) compared to milder winters. No association was found between extreme cold months and neonatal mortality for the non-Sami population. Warm months (+15.1°C) had no impact on Sami or on non-Sami neonatal mortality risk. We note a couple of possible explanations for the decline in cold related neonatal mortality among the Sami after 1860. First, in rural northern Sweden until the late nineteenth century, the delivery of infants was mainly a private matter in one's home and visiting a doctor was a rare occasion [5], especially true among the Sami [7]. At the end of the 1800s the presence of trained midwives increased, preventing the risk of Sami neonatal mortality during cold extremes, especially due to hypothermia. Second, the Sami experienced overall improvements in living standards and health status, which contributed to a decline in weather vulnerability.

Compared to other studies of the association between seasonality and neonatal mortality among historical populations [11,19], neonatal mortality in Sápmi did not reveal a pronounced U-profile of neonatal mortality by month of birth. This can be explained by the differences in climate between northern Sweden and the Mediterranean countries, in which the former has shorter and cooler summers and longer, colder winters. In northern Sweden, daily average temperatures below 0°C are common from October to April, making the winter (meteorological) a very long season compared to southern Europe. The combination of long winters and adaptation to a harsh climate are plausible factors why seasonal differences in neonatal mortality are less pronounced than in Mediterranean countries. However, for the Sami, neonatal mortality was significantly higher during the colder months of January and February, whereas there was less variation in mortality by month among the non-Sami population. This higher neonatal mortality during the cold winter among the Sami could relate to the hazardous nature of reindeer herding and not exclusively to the movements between the settlements, as well as to wood smoke exposure from the huts, which might have enhanced the effect of low temperatures for neonatal mortality. The neonatal mortality rate also increased among Sami infants born in September. Following the annual cycle of reindeer herding, September corresponds to a work-intensive period with the slaughtering of reindeer and milking of cows. During the summer months (June–August), Sami women with infants or who were pregnant had the least work-intensive period [45]. This shift in work intensity among Sami women is supposed to be associated with the increase in September in neonatal mortality. We know that winter was generally a difficult season for all populations in northern Sweden during the nineteenth century (cold weather, snow, infectious diseases and disruptions of the food supply) [46]. This study has revealed that the Sami neonatal mortality rate was generally higher in the winter compared to other seasons and that more Sami infants died during extreme cold winters, while non-Sami were less vulnerable to cold.

### Strengths and limitations

To the best of our knowledge, this study is the first of its kind to investigate the association between extreme temperature and neonatal mortality in a pre-industrial sub-Arctic environment using temperature recordings and historical population data. The temperature data were based on measurements from Tornedalen in the northern part of Sápmi and are a good representation of weather exposures for the majority of the population in this study. A limitation is that temperature data are based on monthly mean temperatures instead of daily mean or minimum temperature. Compared to previous studies of neonatal mortality using daily temperatures [11] that revealed the effect of temperature on the day of birth, the present study provides a rough picture of the association between cold weather extremes during the month of birth and neonatal mortality.

### Conclusions

This study concludes that the cold extremes affected neonatal mortality only in the Sami population. We revealed the role of environmental factors for infant mortality during the demographic and socio-economic transitions of the nineteenth century and highlighted the population most at risk. In a global perspective of today, children and infants are especially vulnerable to weather extremes [47,48]. Our findings of the association between cold extremes and neonatal mortality among two ethnic groups might function as an eye-opener of patterns behind climate-related ill-health among in resource-poor rural communities in low-, middle-, and high-income countries.

## Paper context

There is a limited number of studies regarding the association between weather extremes and neonatal mortality in pre-industrial sub-Arctic societies. We found that the Sami population was most at risk of cold extremes, whereas warm extremes had no impact on Sami or non-Sami neonates. Research of climate vulnerability among populations sharing the same environment and climate but diverging in cultural and economic practices may be an eye-opener of patterns behind climate-related neonatal mortality across time and place.
